# Network Hyperexcitability in Early Alzheimer’s Disease: Is Functional Connectivity a Potential Biomarker?

**DOI:** 10.1007/s10548-023-00968-7

**Published:** 2023-05-12

**Authors:** C. J. Stam, A. M. van Nifterick, W. de Haan, A. A. Gouw

**Affiliations:** 1 Department of Neurology, Amsterdam Neuroscience, Clinical Neurophysiology and MEG Center, Vrij Universiteit Amsterdam, Amsterdam UMC, PO Box 7057, 1007 MB Amsterdam, The Netherlands; 2 Alzheimer Center Amsterdam, Neurology, Vrije Universiteit Amsterdam, Amsterdam UMC location VUmc, Amsterdam, The Netherlands; 3 Amsterdam Neuroscience, Neurodegeneration, Amsterdam, The Netherlands

**Keywords:** Functional connectivity, E/I balance, Brain networks, Computational model, EEG, MEG, Mild cognitive impairment, Alzheimer’s disease

## Abstract

Network hyperexcitability (NH) is an important feature of the pathophysiology of Alzheimer’s disease. Functional connectivity (FC) of brain networks has been proposed as a potential biomarker for NH. Here we use a whole brain computational model and resting-state MEG recordings to investigate the relation between hyperexcitability and FC. Oscillatory brain activity was simulated with a Stuart Landau model on a network of 78 interconnected brain regions. FC was quantified with amplitude envelope correlation (AEC) and phase coherence (PC). MEG was recorded in 18 subjects with subjective cognitive decline (SCD) and 18 subjects with mild cognitive impairment (MCI). Functional connectivity was determined with the corrected AECc and phase lag index (PLI), in the 4–8 Hz and the 8–13 Hz bands. The excitation/inhibition balance in the model had a strong effect on both AEC and PC. This effect was different for AEC and PC, and was influenced by structural coupling strength and frequency band. Empirical FC matrices of SCD and MCI showed a good correlation with model FC for AEC, but less so for PC. For AEC the fit was best in the hyperexcitable range. We conclude that FC is sensitive to changes in E/I balance. The AEC was more sensitive than the PLI, and results were better for the thetaband than the alpha band. This conclusion was supported by fitting the model to empirical data. Our study justifies the use of functional connectivity measures as surrogate markers for E/I balance.

**Supplementary Information:**

The online version contains supplementary material available at 10.1007/s10548-023-00968-7.

## Introduction

Network hyperexcitability (NH) is a signature of a disturbed balance between excitation and inhibition (E/I balance) and is increasingly considered to be a key feature in the pathophysiology of Alzheimer’s disease (AD). Early studies in animal models of AD demonstrated a direct relation between activity of synapses and levels of amyloid beta deposition in the surrounding interstitial fluid (Cirrito et al. 2005; Maestú et al. 2021; Tombini et al. 2021). It has become clear that many animal models of AD show a high incidence of epileptic seizures and interictal epileptiform discharges (for review see: Cope et al. 2022; Palop and Mucke 2007; Tok et al. 2022). The network hyperexcitability in AD animal models can be caused by abnormal depositions of amyloid beta and tau, but can also promote further depositions of these abnormal proteins (Tombini et al. 2021; Wu et al. 2016). There are also indications that manifestations related to epilepsy are especially prevalent during sleep, which could guide hypotheses concerning possible underlying mechanisms (Szabo et al. 2022).

The observation of network hyperexcitability in animal models of AD has raised the question whether NH can also be detected in human subjects with AD, especially in the early stages, and whether this could point the way towards possible new approaches for treatment (Altuna et al. 2022; Babiloni 2022; Horvath et al. 2016; Vossel et al. 2017). Several studies have shown that AD patients have a higher risk of suffering from epileptic seizures and a higher prevalence of interictal epileptiform discharges (for review see: Csernus et al. 2022). There are also indications that epileptic seizures in AD are often subclinical, and may require special techniques such as foramen ovale electrodes for discovery (Lam et al. 2017, 2019). In a large retrospective study of routine EEG recordings in a memory clinic, subclinical epileptiform discharges were detected in 3% of all subjects and across different types of dementia, and these subjects were significantly younger (Liedorp et al. 2010). Vossel et al. (2016) used resting-state magnetoencephalography (MEG)/electroencephalography (EEG) in combination with all night ambulatory EEG recordings and found subclinical epileptiform discharges in 42.4% of AD subjects compared to 10.5% of healthy controls. Presence of epileptiform discharges was associated with a more rapid progression of cognitive dysfunction. In a study with ambulatory EEG Lam et al. (2020) reported a prevalence of interictal epileptiform abnormalities in 53% of AD patients with a known history of epilepsy, 22% of AD without such history, and 4.7% of healthy controls. Babiloni et al. (2020) reported interictal abnormalities in 41% of subjects with MCI, and suggested a relation with more abnormalities in the delta band. In a more recent study this group reported a relation between interictal epileptiform discharges and increased slowing in subjects with MCI due to AD pathology (Babiloni et al. 2022). In a study with 24 h ambulatory EEG recordings subclinical epileptiform discharges were detected in 54% of AD patients and 25% of control subjects (Horvath et al. 2021). Epileptiform activity was associated with worse cognition and more rapid progression. However, another study using sleep EEG recordings did not report a significant increase in epileptiform abnormalities in AD or mild cognitive impairment (Brunetti et al. 2020).

Network hyperexcitability in AD is not only important for understanding the underlying disease mechanism, but also as a possible target for treatment (Canter et al. 2016a, b). A recent small clinical trial could not show an overall effect of treatment with an anti-epileptic drug (levetiracetam) on cognition in AD patients, but a subset of AD patients with subclinical epileptiform discharges did show improvement on a test of executive functioning and a test of virtual route learning (Vossel et al. 2021). Overall, these clinical studies point to an increased incidence of phenomena related to NH such as seizures and interictal epileptiform discharges, possibly clinically relevant in early treatment, but the estimates of the prevalence vary widely, and there are large methodological differences between the studies. This suggests that larger studies are necessary, and that more reliable biomarkers of NH may be needed (Luppi et al. 2022; Yu et al. 2021).

One candidate biomarker for network hyperexcitability in AD is functional connectivity: the correlation between activity recorded from different brain regions. AD is characterized by a decrease in functional connectivity in higher frequency bands, and an increase in connectivity in the theta band (Schoonhoven et al. 2022). Changes in functional connectivity are correlated with depositions of pathological proteins (Ranasinghe et al. 2020, 2021). Recently it has been shown that Alzheimer patients with interictal epileptiform abnormalities have lower functional connectivity in the alpha band and higher connectivity in a combined delta theta band compared to AD patients without epileptiform abnormalities (Ranasinghe et al. 2022). A loss of functional connectivity in the gamma band has also been described in MCI subjects with interictal epileptiform abnormalities (Cuesta et al. 2022a, b).These studies suggest that functional connectivity (FC) could be an alternative biomarker for network hyperexcitability in AD. If it could be shown that FC is more sensitive and/or reliable than interictal epileptiform discharges, it could be used in clinical trials to select patients who are more likely to benefit from treatment with anti-epileptic drugs.

However, FC can be assessed with different types of measures, and in different frequency bands, and it is unclear how these are related to the excitation inhibition balance of the underlying networks. In particular, measures of amplitude correlations and measure of phase synchronization are often used to assess FC of EEG and MEG recordings, but these may reflect different intrinsic coupling modes (ICM) of brain networks, and cannot easily be reduced to each other (Engel et al. 2013; Siems and Siegel 2020).

These types of questions can be addressed with computational models of whole brain networks (for review see: Stefanovski et al. 2021). In an early simulation study, we showed how an activity dependent degeneration (ADD) scenario, where high levels of synaptic activity would induce weakening of synaptic strength, could explain spectral slowing, loss of functional connectivity (after an initial increase in the MCI phase), and selective involvement of highly connectivity hub nodes (de Haan et al. 2012). This model has also been used to investigate the impact of different therapeutic interventions (de Haan et al. 2017). Demirtas et al. (2017) used empirical structural and functional MRI data and a large-scale brain network model of coupled Stuart Landau oscillators to show that progressive stages of AD are characterized by an increased distance from the putative ideal, critical state with optimal E/I balance. In a very extensive study, using the computational framework of the virtual brain, Stefanovski et al. (2019) showed how local changes in hyperexcitability of neural masses, informed by individualized information about amyloid beta deposition based upon PET recordings, could be used to reproduce empirical findings in AD such as region-specific spectral slowing. In addition, the effect of treatment with an NMDA antagonist could be studied. Tait et al. (2021) used individualized brain models based upon empirical observed functional connectivity to study the propensity for developing epileptic seizures. They observed an increased brain network ictogenecity (BNI) in the AD group. These large-scale model studies in AD clearly show the potential of this approach to address questions with respect to pathophysiological mechanisms and their implications. However, none of these studies specifically addressed how changes in the E/I balance would affect FC as assessed by measures of amplitude correlation versus measures of phase synchronization in different frequency bands. This type of information is important since it could guide the choice of optimal biomarkers to select AD patients with high NH in clinical trials.

In the present study we use a computational whole-brain model of structural and functional brain networks to address the following two questions: (i) how does the EI balance, in combination with the connectivity strength, affect the FC as measured by amplitude correlation or phase synchronization metrics? (ii) can we use empirical FC networks of subjects with subjective cognitive decline and subjects with mild cognitive impairment in theta and alpha bands to obtain consistent estimates of the EI balance of the underlying networks?

## Methods and Materials

### Model Simulation and Signal Analysis

#### Brain Dynamics: Stuart Landau Oscillator

**Fig. 1:**
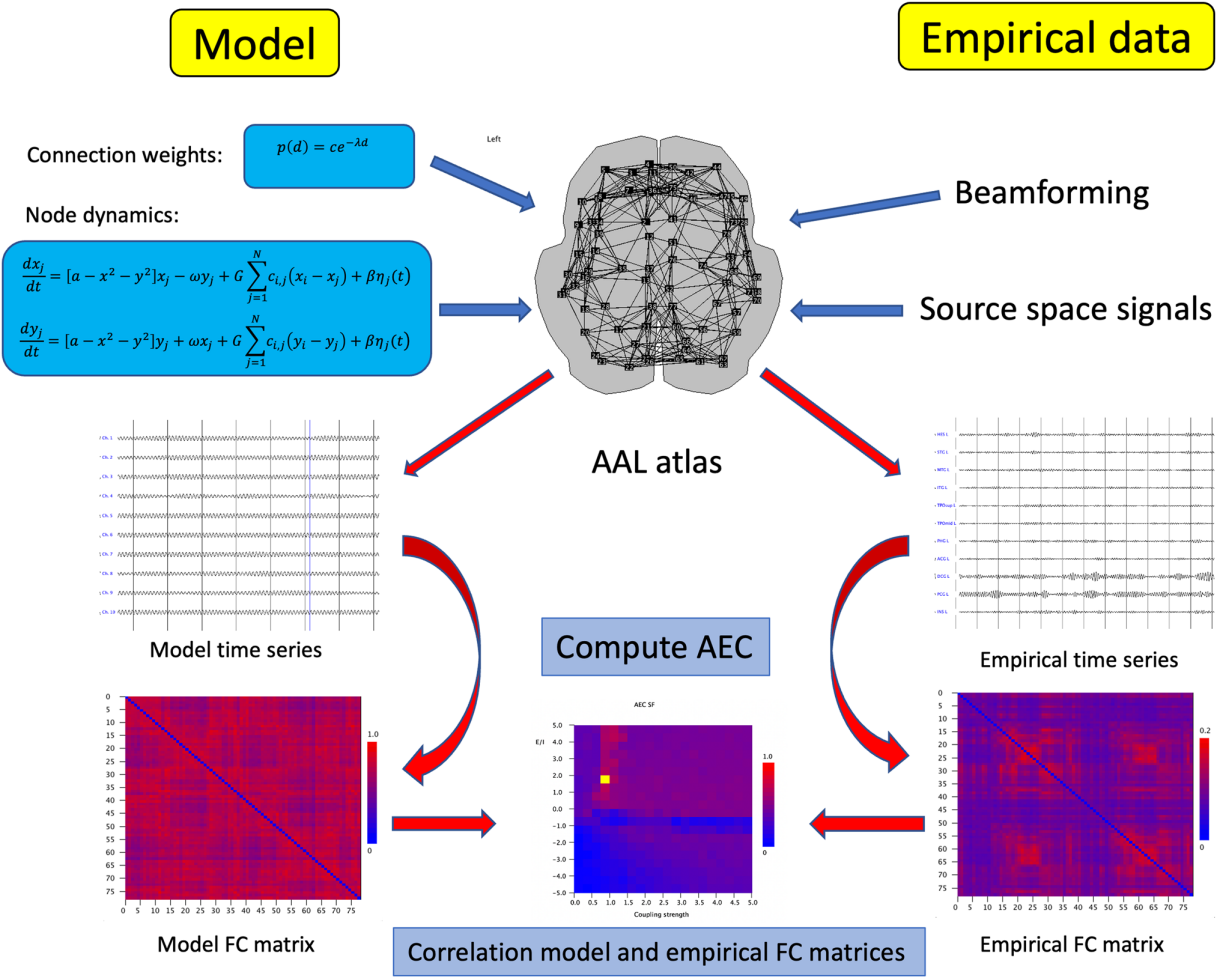
Schematic overview of the methods. For both the model as well as the empirical MEG recordings we reconstructed activity at the centroids of 78 cortical ROIs of the AAL atlas, shown at the top in the middle column. In the model connection weights between all 78 ROIs were based upon an exponential distance rule. Dynamics of the nodes was simulated with a Stuart Landau model, with a slightly different frequency for each ROI. Model time series were generated for all ROIs. From these multichannel data the amplitude envelope correlation was computed, resulting in a model empirical functional connectivity matrix. For the empirical MEG data, signals were filtered in the alpha band (8–13 Hz) or the theta band (4–8 Hz), and projected to the centroids of the 78 cortical ROIs using beamformer techniques. This resulted in 78 channels of source reconstructed MEG data. From these the AEC was computed, with pairwise correction for volume conduction (AECc). This resulted in an empirical functional connectivity matrix. Finally, the correlation between model and empirical FC matrices was computed as a function of the coupling strength G in the model (bottom, middle column)

**Fig. 2:**
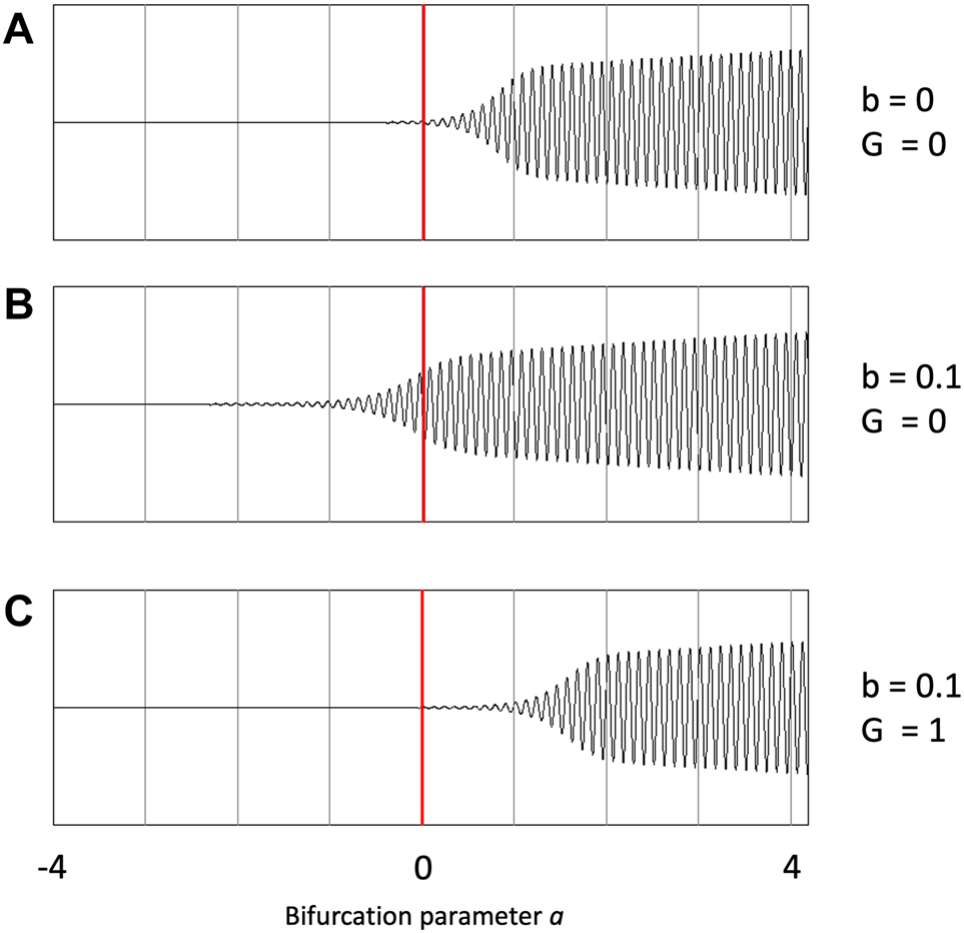
Change in model time series under influence of a, noise and coupling G. **A **Model time series of a single channel as a function of bifurcation parameter a (from − 4 to 4), for a noise level of b = 0 and no coupling (G = 0). The bifurcation parameter can be interpreted as a crude approximation of the excitation/inhibition balance in the model. Note the transition between a flat line to oscillatory fluctuations at the bifurcation point a = 0. **B **Same data, but now for a noise level of b = 0.1 and G = 0. Note that the addition of noise results in the blurring of the transition point. **C** Same data, but now for b = 0.1 and G = 1. Note that adding coupling to the model influences the onset of oscillations, which now occur at a positive value of *a*

To simulate oscillatory activity of the local nodes in the whole brain network model we used the Stuart Landau model, also referred to as the Hopf bifurcation model. An overview of the model and the setup is shown in Fig. 1, and a summary of model parameters and their interpretation is shown in Table 1. This is one of the simplest models which has phase as well as amplitude information. This is important, since we want to study the behavior of functional connectivity measures which depend upon amplitude envelope correlations (AEC) as well as measures which depend upon phase differences between oscillators. The model has a bifurcation parameter *a* which controls a phase transition between a regime with no oscillations (point attractor) and a regime with oscillations (limit cycle). This parameter is of particular interest for the present study since it can be interpreted as a crude description of the E/I balance. The influence of the bifurcation parameter *a* on the model time series is shown in Fig. 2. The model has been used extensively to simulate brain dynamics in whole brain models of fMRI BOLD signals (Moon et al. 2015; Deco et al. 2017b, 2021; Demirtas et al. 2017; Goriely et al. 2020) and MEG signals (Deco et al. 2017a). The model dynamics is given by the following differential equation:

1 $$\frac{dz_j}{dt}=z\left[a_j+i\omega_j-\left|z_j^2\right|\right]+\beta\eta_j\left(t\right).$$

**Table 1:** Parameter settings of the model

Parameter	Interpretation	Value	Range
ω	Frequency	6,10 Hz	
	Frequency range	1 Hz	
	Sample frequency	500 Hz	
	Euler integration step	0.002	
	Epoch length	4096 samples	
a	Bifurcation parameter (excitation/inhibition balance)		− 5–5
b	Noise level	0.1	
η	Gaussian white noise	Mean = 0 SD = 1	
G	Global coupling strength		0–5
c_ij_	Local coupling strength		0–1
λ	Exponent exponential distance rule	10	
N	Number of nodes (AAL atlas)	78	

Here (in formula 1) z is a complex number, *a* is the bifurcation parameter, ω is the frequency (in radians/second), β is the noise level, and η is Gaussian white noise with zero mean and a standard deviation of 1. In the present study we used a noise level of β = 0.1, unless stated otherwise. The mean frequency ω was 6 or 10 Hz, depending on the frequency band of interest (theta or alpha), with a variation of + or – 0.5 Hz around the center frequency. By considering the real part of z (x) and the imaginary part (y) separately we can write (Deco et al. 2017b):

2 $$\frac{dx_j}{dt}=\left[a-x^2-y^2\right]x_j-\omega y_j+G\sum_{j=1}^Nc_{i,j}\left(x_i-x_j\right)+\beta\eta_j\left(t\right),$$

3 $$\frac{dy_j}{dt}=\left[a-x^2-y^2\right]y_j+\omega x_j+G\sum_{j=1}^Nc_{i,j}\left(y_i-y_j\right)+\beta\eta_j\left(t\right),$$

Here i and j are indices of different oscillatory systems, G is a global parameter of structural coupling strength, and c_i,j_ is the weight of the structural connections between dynamical system i and j. As can be seen in [2] and [3] G and c_i,j_ are multiplied to obtain the obtain the structural connectivity strength. The variable x is taken as the oscillatory output of the system. By writing the system equations in this way the instantaneous phase and amplitude can be obtained immediately:

4 $$\phi_t=arctan\frac{y_t}{x_t},$$

where ϕ_t_ is the phase at time t (in radians) and

5 $$amplitude\,envelope_{t} = \sqrt {x_{t}^{2} + y_{t}^{2} }$$

is the amplitude envelope or instantaneous amplitude at time t. We use Euler’s method for numerical integration of the model with a step size of 0.002 and a sample frequency of 500 Hz. Model output is in the form of trials or epochs of 4096 datapoints, each preceded by 5000 time steps to get rid of transients at the start of each trial in order to discard any transients in the dynamics.

#### Structural Brain Networks

We constructed a network with 78 connected oscillators. The locations of the 78 oscillators corresponded with the coordinates of cortical regions of interest of the AAL atlas (Tzourio-Mazoyer et al. 2002). This atlas was chosen to allow comparison of the functional connectivity matrices with the empirical MEG recordings described below. To obtain the weights of the structural connections between all 78 nodes we used the exponential distance rule (Ercsey-Ravasz et al. 2013).

6 $$p\left(d\right)=e^{-\lambda d}$$

Here *p(d)* is the probability that two regions separated by a Euclidian distance *d* are connected. We normalized d by dividing it by the largest distance between any two oscillators. We used λ = 10 based upon exploratory studies (Supplementary Fig. 1). All connection weights were divided by the highest weight in the matrix to obtain the connection weights c_i,j_ of eqs. (2) and (3). Structural networks generated with this exponential distance rule closely resemble the ground truth of anatomical connectivity derived from tract-tracing studies in animals (Ercsey-Ravasz et al. 2013) and have been shown to correlate strongly with MRI tractography-based networks (Deco et al. 2021). (Supplementary Fig. 2).

#### Characterization of Amplitude Envelope Correlation and Phase Synchronization

**Fig. 3:**
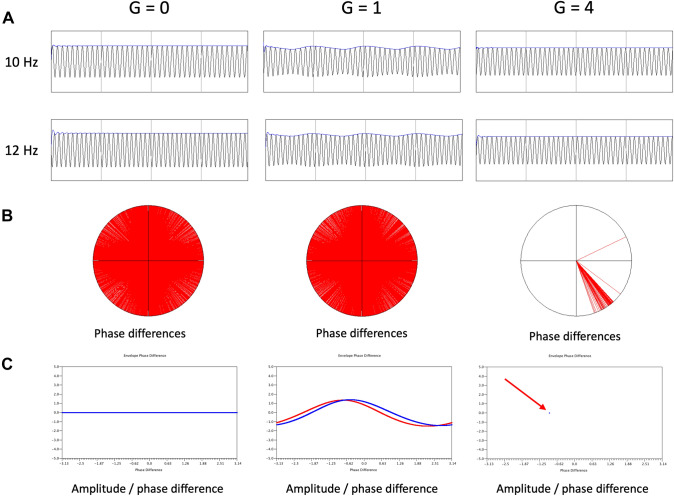
Results for two coupled non identical oscillators. **A** Time series of two oscillators, one with a frequency of 10 Hz (upper row) the other with a frequency of 12 Hz (lower row) as a function of coupling strength G (0, 1 and 4). The amplitude envelope of the timeseries is shown on top in blue. Note that amplitude envelope fluctuations appear only for G = 1. **B** Distribution of phase differences between the two oscillator time series on the unit circle for different coupling strengths G (0, 1 and 4). For G = 0 and G = 1 the phase difference takes on all possible values, whereas for G = 4 the phase difference is limited to a very small range. **C** Plot of normalized (z-scores) amplitude envelopes of the time series of the two oscillators as a function of phase difference G (0, 1 and 4). For G = 0 there are no amplitude envelope fluctuations, but the phase differences take all possible values. For G = 1 there are clear amplitude envelope fluctuations, which are closely related for the two oscillators (amplitude envelope correlation); the phase difference still takes on all possible values (no phase synchronization). For G = 4 there are no longer amplitude fluctuations, but the phase difference is restricted to a very small range (phase synchronization)

Our aim was to determine the relation between functional connectivity measures and the E/I balance in the underlying networks. Here we choose to investigate two measures which are representative for broad categories of functional connectivity: (i) amplitude envelope correlation (AEC) and (ii) phase coherence (PC). Amplitude envelope correlation and phase coherence are illustrated schematically for two coupled oscillators with different frequencies in Fig. 3. The choice to study these two coupling measures is motivated by the fact that amplitude correlations and phase coherence/synchronization probably reflect two fundamentally different modes of communication in brain networks (Engel et al. 2013; Qiu et al. 2020; Schoonhoven et al. 2022; Siems and Siegel et al. 2020). The amplitude envelope correlation is the Pearson correlation coefficient of the amplitude envelopes of pairs of channels, which can be obtained directly from the model as shown in formula [4]. We only used positive values of the Pearson correlation coefficient. Negative values were set to zero. Phase coherence between all pairs of channels was computed as follows:

7 $${phase\,coherence}_{i,j}=\left|\sum_{t=1}^Te^{i\varphi _{i,j}\left(t\right)}\right|/n,$$

where i and j indicate oscillators, t is discrete time, T is the total number of time steps (4094 per epoch) and ϕ_i,j_ the instantaneous phase difference between oscillator i and j at time t. The phase difference can be obtained directly from the model output using formula 4. In the model functional connectivity was computed for all pairs of channels and for each trial/epoch of 4096 samples. The resulting functional connectivity matrices were subsequently averaged over multiple runs to obtain a better signal-to-noise ratio.

To compute amplitude envelope correlation and phase synchronization from empirical datasets it is necessary first to extract the instantaneous phase from the recorded signals, and next to correct for the influence of volume or field spread. As described previously (Stam et al. 2007), this can be realized using the analytical signal based on the Hilbert transform. The analytical signal z_t_ is complex-valued with x_t_ a real time series and ${\tilde{x}}_{t}$ its corresponding Hilbert transform:

8 $$z_t=x_t+i{\widetilde x}_t=A_te^{i\varphi_t}.$$

The Hilbert transform of x_t_ is obtained via integration as follows:

9 $${\widetilde x}_t=\frac1\pi PV\int_{-x}^\infty\frac{x_t}{t-\tau}d\tau,$$

where PV refers to the Cauchy principal value. Note that x_t_ corresponds to the x in Eq. (2), and $i{\tilde{x}}_{t}$to the y in Eq. (3). The Hilbert transform [9] is related to the original signal by a [1/2]π phase shift that does not alter the spectral distribution (it can be computed by performing a Fourier transform, shifting all the phases by [1/2]π, followed by an inverse Fourier transform). From Eq. (8), both the instantaneous amplitude A_t_ and the instantaneous phase ϕ_t_ can be obtained in a similar way as in (4) and (5). For the computation of functional connectivity measures (amplitude envelope correlation as well as phase synchronization) from empirical data the influence of volume conduction or field spread has to be dealt with as well. In the case of the amplitude envelope correlation this is accomplished by pair-wise orthogonalization of the data before the AEC is computed (Hipp et al. 2012; O’Neill et al. 2015; Schoonhoven et al. 2022). We refer to this corrected version of the AEC as the AECc. In the case of phase synchronization, we used the phase lag index (PLI) which is not sensitive to volume conduction (Stam et al. 2007):

10 $${PLI}_{i,j}=\left|\langle sign\left[\text{sin}\left(\varphi_{i,j}\right)\right]\rangle\right|.$$

Here *sign* is the signum function which returns 1 if the argument if positive and − 1 otherwise, and ϕ_i,j_ is the instantaneous phase difference between oscillators i and j.

### Empirical Data

#### Subjects

In the present study we re-analyzed a dataset which was previously used in two other studies for different analyses focused on spectral power (Luppi et al. 2022) and symbolic dynamics (Scheijbeler et al. 2022). As previously described (Scheijbeler et al. 2022) the study involved two age- and gender-matched groups consisting of 36 subjects: 18 subjects with subjective cognitive decline (SCD; mean age 64.2 years, SD 6.1; 8 males; MMSE 27.8, SD 2.1) and 18 subjects with amnestic mild cognitive impairment (MCI; mean age 64.1 years, SD 6.2; 9 males; MMSE 25.8, SD 1.9). The data was retrospectively collected from the Amsterdam Dementia Cohort (Van der Flier and Scheltens 2018), which included data of subjects who visited the memory clinic of the VUmc Alzheimer Center in the period of spring 2015–2018 and provided written informed consent for the use of their data for research purposes. As part of an extensive diagnostic work-up, all subjects received medical history taking, neurological and neuropsychological examination, blood tests, 3T MRI of the brain, routine MEG and, if possible, a lumbar puncture to collect cerebrospinal fluid (Van der Flier and Scheltens 2018). A multidisciplinary team decided upon a diagnosis during a consensus meeting and according to the 2011 National Institute on Aging-Alzheimer’s Association (NIA-AA) criteria. Positive amyloid biomarkers were available for all MCI subjects (by cerebrospinal fluid (CSF) ptau/amyloid ratio > 0.020 and/or abnormal amyloid PET). All SCD subjects, except three subjects with unknown biomarker status, had confirmed negative amyloid biomarkers.

#### MEG Recordings and (Pre-)Processing

MEG recordings were obtained in a magnetically shielded room using a 306-channel whole-head vectorview MEG system (Elekta Neuromag Oy, Helsinki, Finland) at a sample frequency of 1250 Hz, with an online anti-aliasing filter of 410 Hz and high-pass filter of 0.1 Hz. The head position relative to the MEG sensors was continuously recorded and digitized using four to five head-localization coils and a 3D-digitizer (Fastrak, Polhelmus, Colchester, VT, USA). The scalp outline (~ 500 digitized points) was used for coregistration to the best fitting structural MRI template. The sensor-space data was filtered by a temporal extension of the signal space seperation (tSSS) filter (implemented in MaxFilter software, Elekta Neuromag Oy, version 2.2.15) (Taulu and Simola 2006) and a broad-band filter (0.5–70 Hz). Then, an atlas-based beamforming approach (Hillebrand et al. 2012) was applied to reconstruct time-series of neuronal activity. The centroid voxel of each region of the automated anatomical labeling atlas (AAL; Tzourio-Mazoyer et al. 2002) was representing that region (Hillebrand et al. 2016). The sphere that best fit the scalp surface was used as volume conductor model and an equivalent current dipole as source model for computation of the beamformer weights.

#### MEG Time-Series Analysis

Functional connectivity was calculated for the source-level time-series data of the whole-brain and between each ROI of the AAL atlas. For the AECc analysis the first 20 epochs of the first eyes-closed recording were selected. These epochs were down sampled by a factor of four. This resulted in epochs of 4096 samples in length (duration of 13.11 s). For the computation of the PLI no down sampling was used and 80 epochs of 4096 samples (3.2768 s) of the first eyes-closed recording were selected. Computation of AECc and PLI was done with software written by the first author (BrainWave, version 0.9.165.51, available from home.kpn.nl/stam7883/brainwave.html). For the present study the epochs were filtered in two canonical frequency bands, i.e., theta (4–8 Hz), alpha (8–13 Hz) using a discrete fast fourier transform. For both groups and both connectivity measures and both frequency bands grand mean functional connectivity matrices were computed by averaging over all subjects and all epochs. These grand average connectivity matrices were used for comparison with connectivity matrices generated by the model.

## Results

**Fig. 4:**
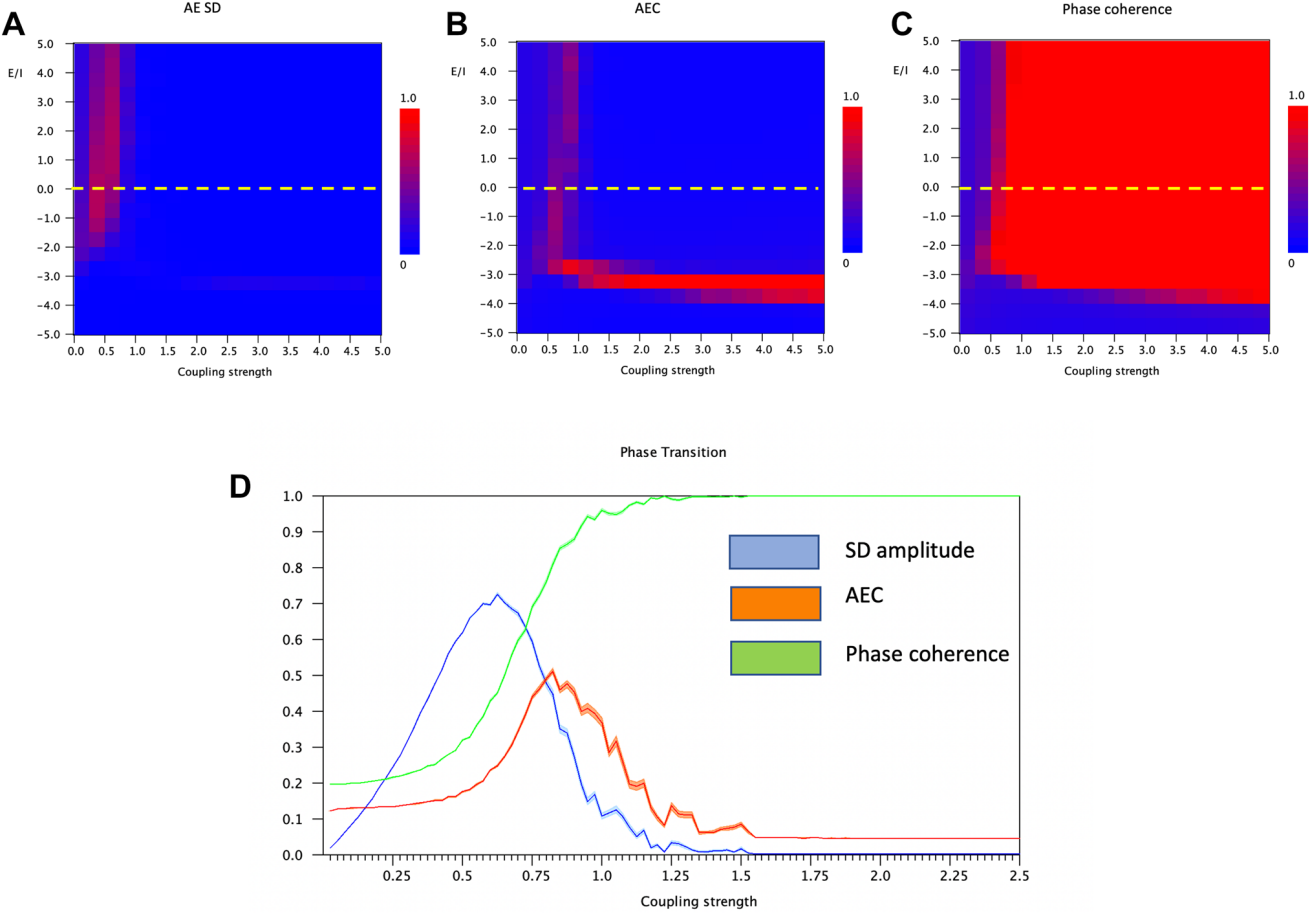
The relation between key model parameters and model output. The oscillators had a mean frequency of 10 Hz (range: 1 Hz) and a noise level of b = 0.1. **A** Standard deviation of amplitude envelope fluctuations per channel as a function of bifurcation parameter a, and coupling strength G.There is a region with a relatively high standard deviation for relatively low G, and positive values of a. **B** Amplitude envelope correlations (AEC) as a function of bifurcation parameter a and coupling strength G. The plot shows two areas with low AEC separated by a narrow boundary with high AEC, determined by a combination of the bifurcation parameter a and the coupling strength G. **C** Phase coherence as a function of bifurcation parameter a and coupling strength G. Phase coherence shows a transition between low and high levels at a transition boundary determined by a combination of the bifurcation parameter a and the coupling strength G. **D **Mean (shaded area: 2 ⋅ standard error of the mean) standard deviation of amplitude envelop per channel (in blue), amplitude envelope correlation (in red) and phase coherence (in green) as a function of coupling strength G for a value of a = 0 (critical point, indicated by yellow line in A, B and C)

As a first step we analyzed how the two functional connectivity measures reflected changes in the excitation inhibition balance, as represented by the bifurcation parameter *a*. An important consideration here is that the effect of the bifurcation parameter on functional connectivity is not independent of the structural coupling strength. For this reason, we studied the mean functional connectivity as a function of both the bifurcation parameter and the structural coupling strength in a single plot. In addition, we also looked at the standard deviation of the amplitude of individual oscillators, since this may also reflect indirectly the consequences of coupling (see Fig. 3, results for coupling strength G = 1). The results are shown in Fig. 4. Figure 4A shows that there is a clear effect of E/I balance and coupling strength on the mean amplitude standard deviation of individual channels. The standard deviation shows a region with large fluctuations for E/I values of bifurcation parameter *a* higher than − 1, up to 5, and for G between 0.25 and 0.75. This suggests that fluctuations of local amplitudes are maximal for relatively high levels of E/I balance, and relatively weak coupling, even before the onset of interregional synchronization.

In Fig. 4B the mean AEC values averaged over all channel pairs and 100 epochs are shown. The plot shows two areas of relatively low AEC separated by a demarcation zone which is determined by the bifurcation parameter and the coupling strength. This pattern suggests that the AEC is maximal at, or close to the onset of interregional coupling. Remarkably, the AEC decreases in the zone with high values of *a* and G. Finally, Fig. 4C shows the results for phase coherence (PC). Here we see a very clear transition between a zone with low PC for low *a* and G, and a zone with very high PC for high *a* and G. The transition between the low and high PC regions seems to coincide with the zone where AEC showed the highest values (Fig. 4B). However, in contrast to the AEC, the PC rapidly increased to a maximum value close to 1, and stays at this high value for high values of *a* and G.

To better appreciate the detailed relationship between local amplitude fluctuations, AEC and PC, we plotted the results for a fixed value of *a* = 0 (bifurcation point, corresponding to interrupted yellow line in plots), as shown in Fig. 4D. Results are the mean of 50 iterations, with 2 × standard error of the mean. Again, we can see that the fluctuations of the local amplitudes reach a maximum at weak coupling, before the onset of interregional synchronization. The AEC reaches a maximum value at the onset of synchronization, whereas the PC only reaches its maximum value for higher levels of coupling. *The first conclusion is that local signal variability and the two functional connectivity measures do reflect changes in the E/I balance, but this effect is different for amplitude dependent and phase related measures, and also depends upon the coupling strength*.

Our second question was whether we can obtain consistent estimates of the E/I balance of the underlying brain networks from empirical resting-state MEG recordings. To address this question, we generated average FC matrices (50 iterations) in the model for different values of the bifurcation parameter *a* and coupling strength G, using either a mean frequency of 6 or 10 Hz (variation around mean frequency + or – 0.5 Hz), and using either AEC or PC as connectivity measure. Next, the Pearson correlation coefficient was computed between these average model matrices and grand average FC matrix of empirical MEG recordings of SCD and MCI groups in the theta (4–8 Hz) or alpha band (8–13 Hz), and using either the AECc or the PLI. We interpreted a high correlation/good fit between empirical FC data and model FC data for specific values of the E/I balance and coupling strength as an indication that the MEG FC data contain information about the E/I balance of the underlying brain networks. A high fit thus would support the potential value of FC as a biomarker of E/I balance.

**Fig. 5:**
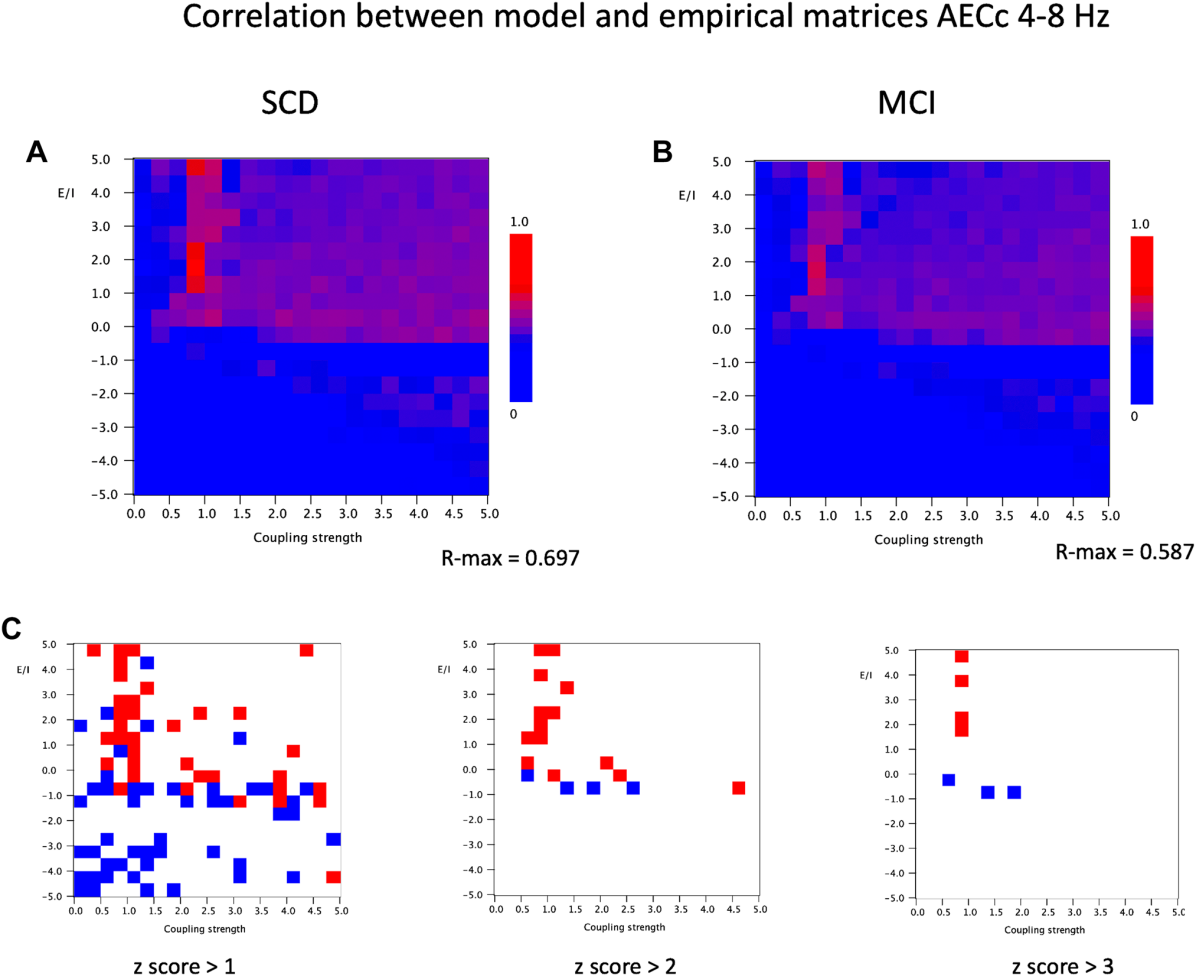
Correlation between model and empirical matrices for AECc in the 4–8 Hz band. **A** Pearson correlation between empirical matrix (AECc in 4–8 Hz band, averaged over 20 epochs and 18 subjects with subjective cognitive decline) and model matrix (mean of 100 iterations; mean frequency 6 Hz) for different combinations of the bifurcation parameter a (from 5 to − 5, in steps of 0.5) and the coupling strength G (from 0 − 5, in steps of 0.25). The strongest correlation between empirical and model matrices is found for positive a, in particular for G around 1. **B** Similar analysis as in A, but now for empirical matrix of 18 subjects with cognitive decline. **C** Differences between matrices shown in A and B. First, the mean and standard deviation were computed for the full 20 × 20 matrices. Next, for each cell, the group difference was expressed as a z-score: [(Pearson matrix A − Pearson matrix B) − mean difference]/standard deviation. Red indicates higher Pearson correlation for the SCD group, blue for the MCI group. Results are shown for three different cutoff levels for the z-score

**Fig. 6:**
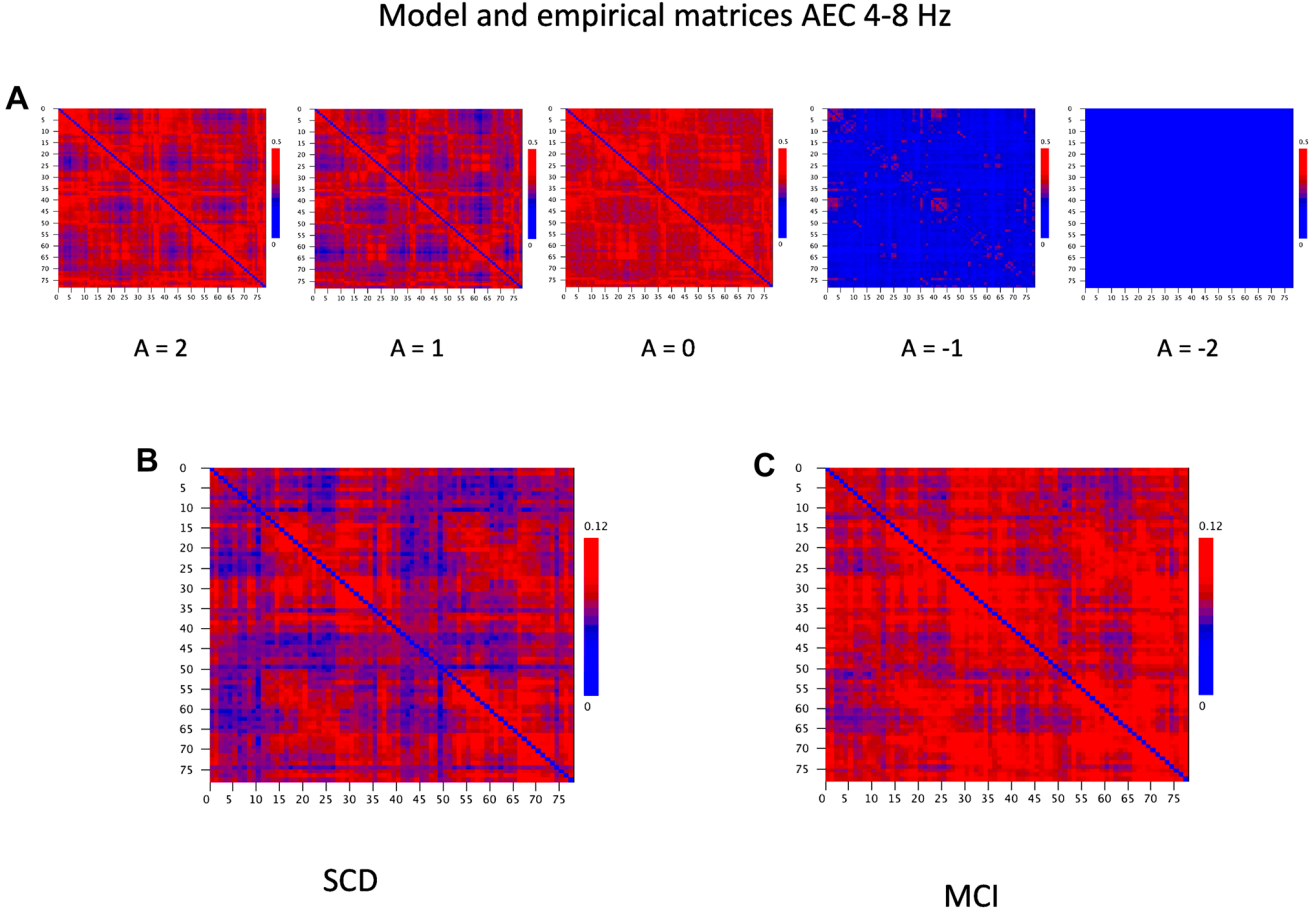
**A** Examples of model AEC matrices at a mean frequency 6 Hz as function of bifurcation parameter a from 2 to − 2 and a fixed noise level b = 0.1 and coupling strength G = 1. **B** MEG AECc connectivity matrix in 4–8 Hz band of subjective cognitive decline (SCD) group, averaged over 20 epochs per subject, and 18 subjects in total. **C** MEG AECc connectivity matrix in 4–8 Hz band of mild cognitive impairment (MCI) group, averaged over 20 epochs per subject, and 18 subjects in total. The functional connectivity matrices of both groups show a greater similarity with model matrices for positive values of the bifurcation parameter a

The results of the empirical model comparison for the AEC/AECc in the theta band are shown in Fig. 5. The correlation plots for the SCD and the MCI groups show a similar pattern with relatively higher empirical model correlations for values of *a* > 0. The highest values are found for a small zone at *a* > 0 and G around 1 (which corresponds roughly with the onset of synchronization; see also Fig. 4). Figure 6 shows the model matrices for this parameter region. Figure 6 suggests that the visual pattern of model and empirical matrices is more informative for positive values of *a* for a coupling strength of 1. The highest Pearson correlation between empirical and model matrices for the SCD group was 0.697. For the MCI group the highest correlation was somewhat lower at 0.587. The region with relatively high empirical model correlation for a > 0 is separated from the lower part of the plots by a blue zone. A second region with relatively high empirical model correlations is found in the lower right part of the plots, but here the correlations are lower than in the upper part.

Although the empirical model correlation plots of the SCD and MCI groups show a very similar organization, they are not identical. To illustrate any patterns in the differences between the two plots we computed the mean and the standard deviation of the differences in Pearson correlation of all 20 × 20 cells. Next, we show the differences in the two matrices expressed as z-scores, with red indicating higher empirical model correlations in the SCD group and blue higher empirical model correlations in the MCI group. The pattern that emerges from Fig. 5C is that the SCD group shows a slightly better model fit for positive values of a (in the high E/I range), especially around G = 1, whereas the MCI group has a somewhat better model fit for values of a at or below zero (in the low E/I range), and for a somewhat broader range of coupling strengths. We should stress however that overall, the similarities in model fit for the SCD and MCI groups are much more striking than the subtle differences.

**Fig. 7:**
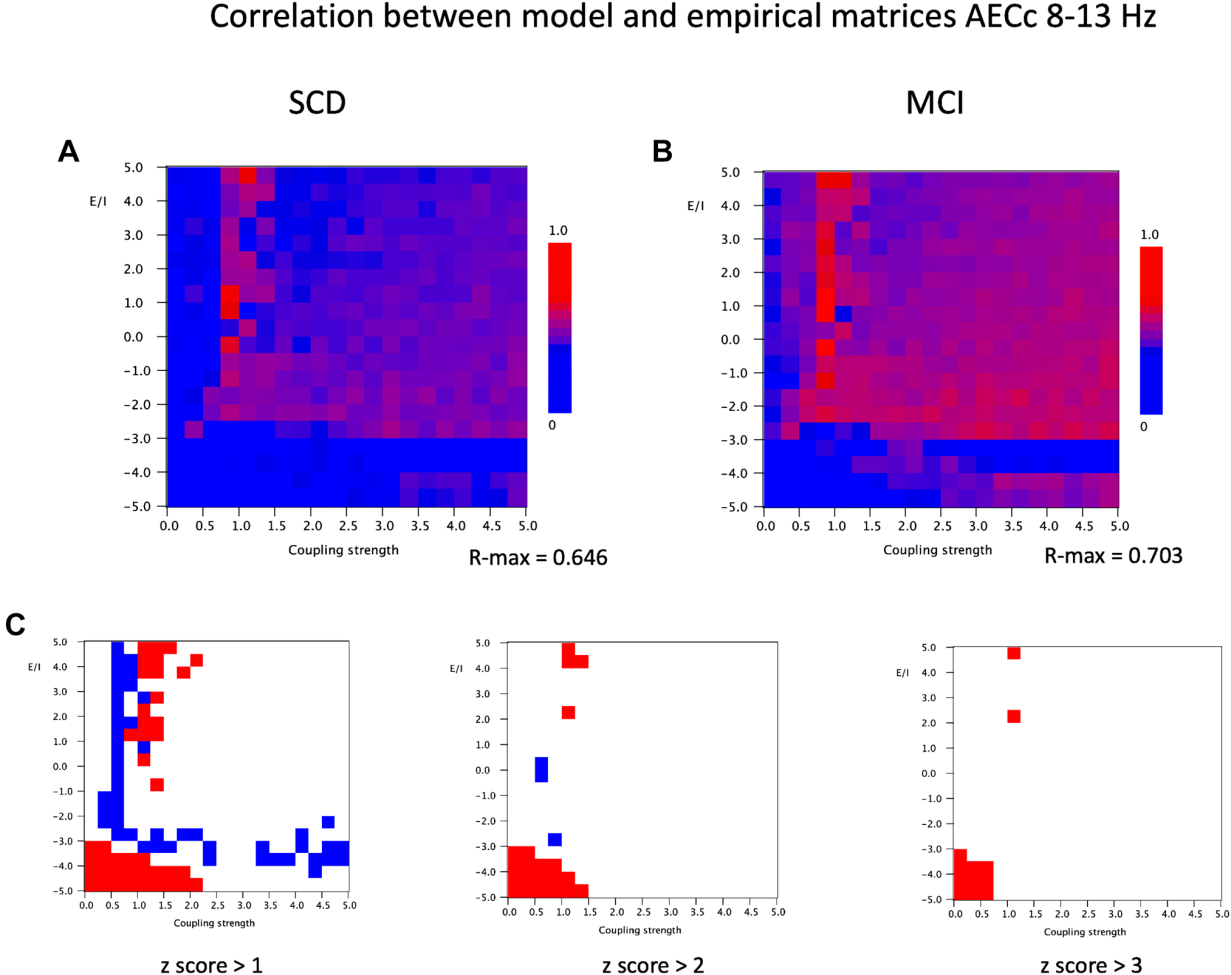
Correlation between model and empirical matrices for AECc in the 8–13 Hz band. **A** Pearson correlation between empirical matrix (AECc in 8–13 Hz band, averaged over 20 epochs and 18 subjects with subjective cognitive decline) and model matrix (mean of 100 iterations; mean frequency 10 Hz) for different combinations of the bifurcation parameter a (from 5 to − 5, in steps of 0.5) and the coupling strength G (from 0–5, in steps of 0.25). The strongest correlation between empirical and model matrices if found for positive a, in particular for G around 1. **B** Similar analysis as in A, but now for empirical matrix of 18 subjects with cognitive decline. **C** Differences between matrices shown in A and B. First, the mean and standard deviation were computed for the full 20 × 20 matrices. Next, for each cell, the group difference was expressed as a z-score: [(Pearson matrix A − Pearson matrix B) − mean difference]/standard deviation. Red indicates higher Pearson correlation for the SCD group, blue for the MCI group. Results are shown for three different cutoff levels for the z-score

A similar analysis was done for empirical and model FC matrices in the alpha band, again using the AEC or AECc. Figure 7 shows that the overall organization of the empirical model correlation plots of both groups is comparable to the results obtained for the theta band. Again, the best model fit is obtained for high values of *a* and a coupling strength around 1, near the phase transition to a synchronized state. The highest Pearson correlation for the SCD group was 0.646, and for the MCI group 0.703. However, one remarkable difference between the theta and the alpha band results is the transition between the upper, high correlation region, and the lower correlation which occurs for the alpha band at a value of *a* around − 3, whereas in the theta band this transition was observed around a value of a around 0. Differences in model fit between the SCD and the MCI group are shown in Fig. 7C. The z-scores show that the SCD group has two regions in parameter space where it has a slightly better model fit than the MCI group: the lower left corned (low *a* and low G), and the region of *a* > 0 and G around 1. Remarkably, the MCI has a better model fit in a narrow zone with *a* > − 3 and G around 0.75, continuing in a zone with *a* around − 3 and G > 0.75. Again, we should stress however that overall, the similarities in model fit for the SCD and MCI groups are much more striking than the subtle differences.

**Fig. 8:**
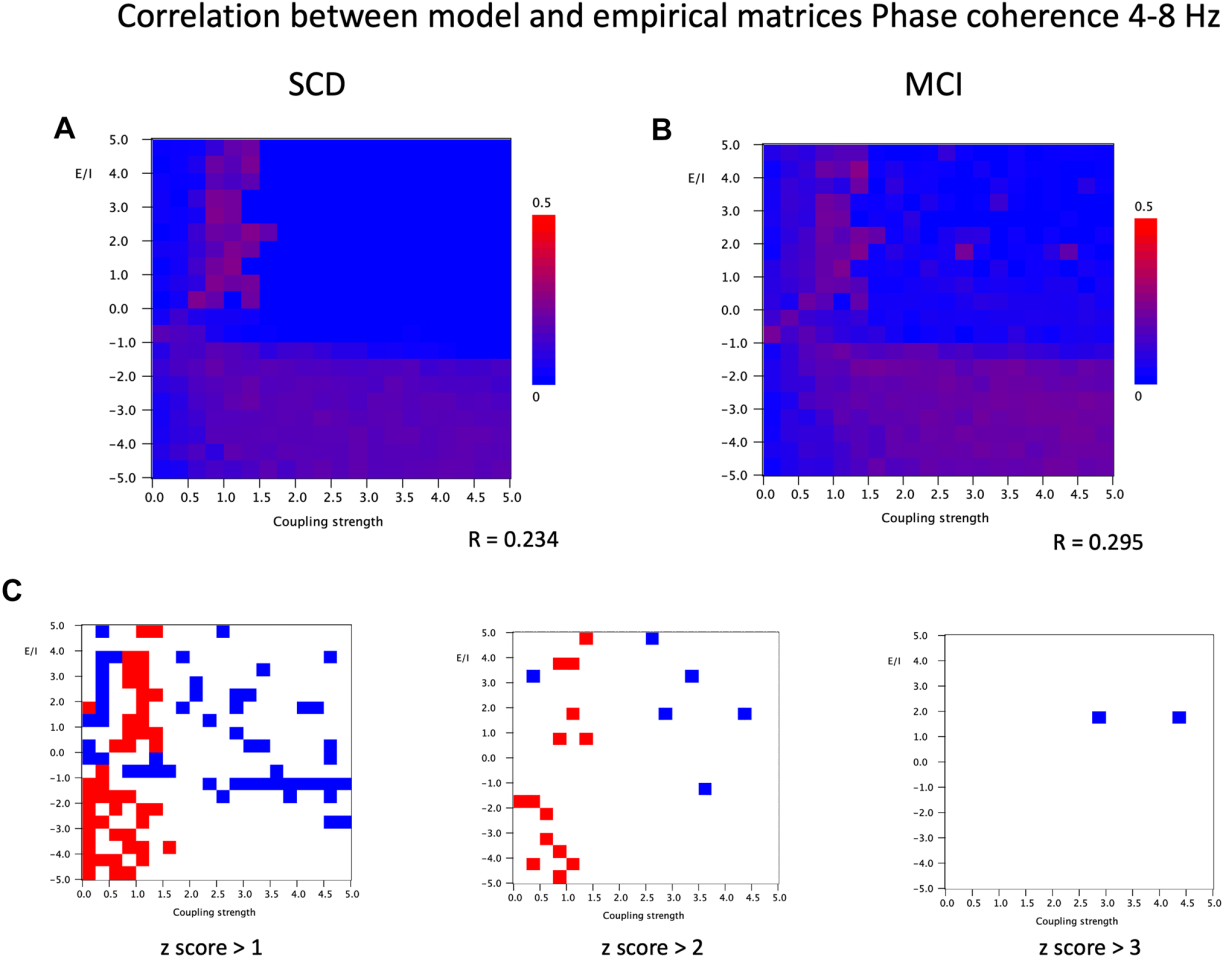
Correlation between model and empirical matrices for phase coherence in the 4–8 Hz band. **A** Pearson correlation between empirical matrix (phase coherence in 4–8 Hz band, averaged over 20 epochs and 18 subjects with subjective cognitive decline) and model matrix (mean of 100 iterations; mean frequency 6 Hz) for different combinations of the bifurcation parameter a (from 5 to − 5, in steps of 0.5) and the coupling strength G (from 0–5, in steps of 0.25). The strongest correlation between empirical and model matrices if found for positive a, in particular for G around 1. **B** Similar analysis as in A, but now for empirical matrix of 18 subjects with cognitive decline. **C** Differences between matrices shown in A and B. First, the mean and standard deviation were computed for the full 20 × 20 matrices. Next, for each cell, the group difference was expressed as a z-score: [(Pearson matrix A − Pearson matrix B) − mean difference]/standard deviation. Red indicates higher Pearson correlation for the SCD group, blue for the MCI group. Results are shown for three different cutoff levels for the z-score

The results of the empirical model functional connectivity for the phase coupling measures (PLI empirical data, phase coherence of model data) for the theta band are shown in Fig. 8. Both SCD and MCI groups show a similar pattern, which is in some respects rather different than the pattern for the AEC. The best model fit is found for a zone of positive *a* around G = 1. However, there is now a large region with *a* < 0 where the model fit is better irrespective of the coupling strength. Also, the overall model fit is substantially worse compared to the results for the AEC. The highest Pearson correlation for the SCD group was 0.234. and for the MCI group 0.295. Due to this very modest model fit differences between the SCD and MCI shown in Fig. 8C have to be interpreted with great care. The SCD shows a better model fit than the MCI group for relatively low G, in particular in the range between 0.25 and 1.5, however this does not seem to depend on the bifurcation parameter *a*. The MCI group shows a better fit with a rather scattered distribution, mainly for *a* > 0, irrespective of G.

**Fig. 9:**
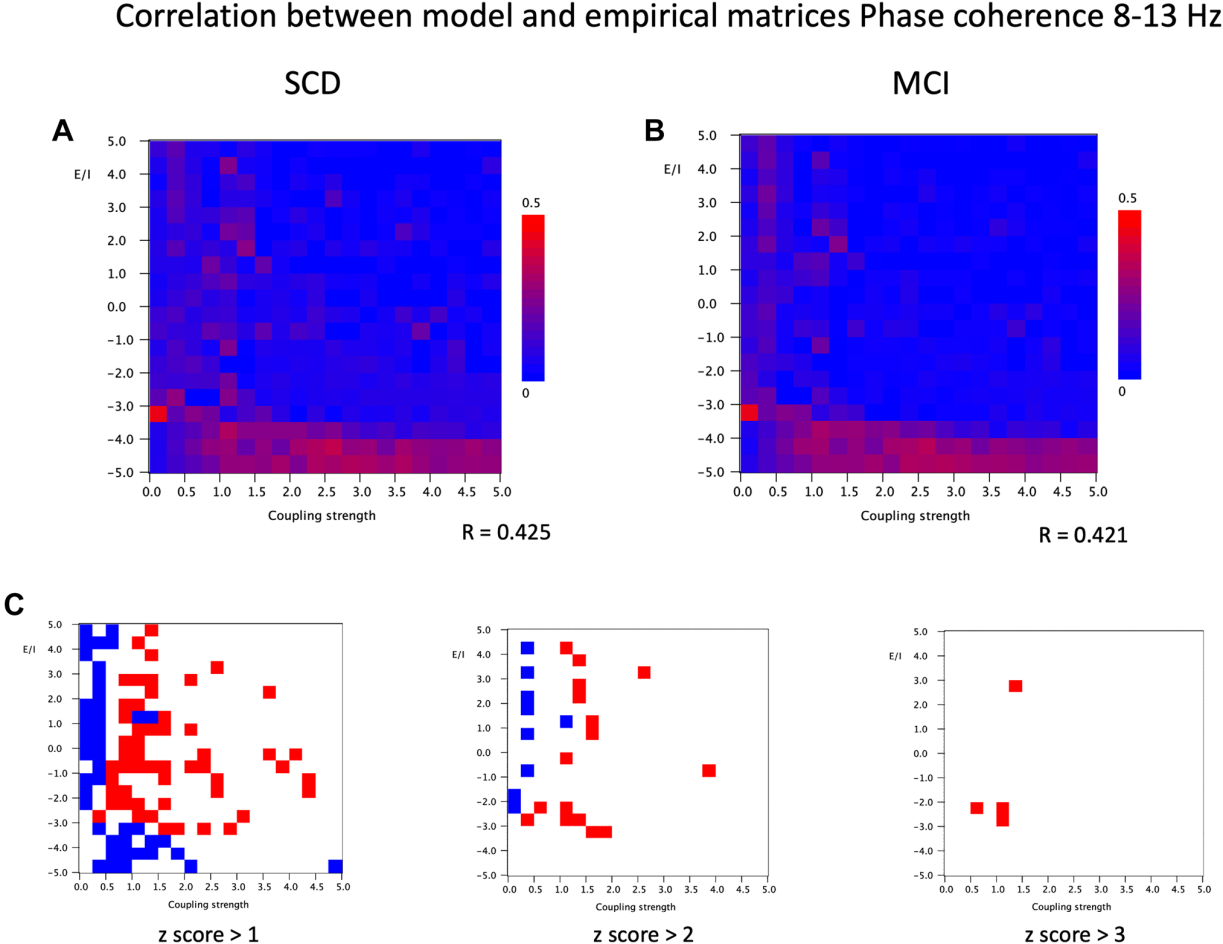
Correlation between model and empirical matrices for phase coherence in the 8–13 Hz band. **A** Pearson correlation between empirical matrix (phase coherence in 8–13 Hz band, averaged over 20 epochs and 18 subjects with subjective cognitive decline) and model matrix (mean of 100 iterations; mean frequency 10 Hz) for different combinations of the bifurcation parameter a (from 5 to − 5, in steps of 0.5) and the coupling strength G (from 0–5, in steps of 0.25). The strongest correlation between empirical and model matrices if found for negative a, in particular for G around 1. **B** Similar analysis as in A, but now for empirical matrix of 18 subjects with cognitive decline. **C** Differences between matrices shown in A and B. First, the mean and standard deviation were computed for the full 20 × 20 matrices. Next, for each cell, the group difference was expressed as a z-score: [(Pearson matrix A − Pearson matrix B) − mean difference]/standard deviation. Red indicates higher Pearson correlation for the SCD group, blue for the MCI group. Results are shown for three different cutoff levels for the z-score

Finally, correlations between empirical and model phase coupling matrices for the alpha band are shown in Fig. 9. In this case there is a striking region of relatively best model fit for both groups for low values of *a*, mostly < − 3.5 to 4, for a broad range of coupling strengths, mostly with G > 0.5. There is a second region with a rather modest model fit in the region with *a* > 0 and G between 0 and 1. The highest Pearson correlation for the SCD group is 0.425 and for the MCI group 0.421. These fits are better than for the theta band, but lower than the fits for the AEC for both bands. Comparison of both groups shown in Fig. 9C shows that the SCD group has a better model fit than the MCI group for slightly higher values of G, over a large range of values of *a*. This shows that both the strength of the model fit, as well as differences in fit between the SCD and MCI groups clearly depend upon the connectivity measure, the frequency band, and a characteristic combination of EI balance and coupling strength.

## Discussion

The present study investigated the relation between E/I balance and functional connectivity measures. Understanding how functional connectivity measures reflect the E/I balance is important if we want to develop reliable biomarkers of network hyperexcitability in Alzheimer’s disease. Those biomarkers could be used for patient selection in clinical trials, and possibly provide information on clinical progression. Our most important conclusion is that both amplitude as well as phase-based connectivity measures were influenced by the excitation inhibition balance. The nature of this dependence was different in both types of measures, and also depended upon the structural coupling strength and the frequency band. Our second conclusion is that it is possible to obtain consistent estimates of E/I balance of the underlying brain networks by fitting the model to empirical resting-state MEG recordings. The best results were obtained for the theta band and the amplitude coupling measure, and suggest that the underlying brain networks are operating in a regime with a high E/I balance near a phase transition.

We showed that both amplitude envelope as well as phase coupling measures reflect E/I balance, but they do so in a different way. In a classic paper the differences between these two families of functional connectivity measures were discussed in detail, and interpreted as two fundamentally different “intrinsic coupling modes” of the brain (Engel et al. 2013). These authors suggested that amplitude correlations might reflect more closely the underlying anatomical connections and the interregional regulation of activity levels, whereas phase synchronization might be an expression of information transfer on relatively short time scales. Recent studies have also confirmed the fundamentally different and non-redundant nature of amplitude and phase-based connectivity measures (Siems and Siegel 2020; Avramieo et al. 2022). Until recently, a proper understanding of the relation between amplitude and phase related measures has been lacking, and the assumption was that amplitude correlations would arise in the context of stronger coupling. However, using the same Stuart Landau model as in the present study, Qiu et al. (2020) were able to show that amplitude envelope correlations can be caused by *weak* coupling between oscillators with slightly different frequencies in the absence of noise and before the onset of phase synchronization.

We have replicated this finding of Qiu et al. (2020) for a simple system of two coupled oscillators (Fig. 3). In the case of a large network of coupled oscillators we also see a different behavior of amplitude and phase coupling: in the parameter plane defined by the excitation–inhibition balance (bifurcation parameter *a*) and the coupling strength G, the AEC showed a local maximum at the transition zone between the non-oscillatory and the oscillatory regimes (Fig. 4B), whereas phase coherence displays a transition from low to high values at approximately the same transition zone (Fig. 4C). This suggests that the AEC is especially sensitive to connectivity in a critical state at the transition between (noisy) point attractor on one side, and a (noisy) limit cycle on the other side. Of interest, a very similar behavior of amplitude envelope correlation and phase synchronization was recently described in a very different model (Avramieo et al. 2022). Importantly, this transition is not a single point, but a demarcation line in the parameter plane determined by both the E/I balance, as well as global coupling strength. In addition to the differences described above, the AEC also showed higher correlations between empirical and model FC matrices. This seems to be related to the fact that amplitude envelope correlations tend to resemble the topology of the underlying structural networks relatively closely, and especially near a bifurcation point in the dynamics (see also Fig. 6). The different behavior of amplitude and phase-based connectivity could perhaps also explain why AEC turns out to be a more robust and reproducible measure in empirical datasets (Colclough et al. 2016; Schoonhoven et al. 2022). Of interest, even very weak coupling in combination with a relatively high excitation inhibition balance could give rise to fluctuations of the amplitude envelopes of individual channels, even before the onset of proper amplitude correlations (Fig. 4D). This observation lends some support to the interpretation of local measures of “neural variability” in terms of the excitation inhibition balance (Garrett et al. 2013; Scheijbeler et al. 2022).

In empirical studies of functional connectivity in Alzheimer’s disease based upon EEG or MEG different and opposite patterns are often observed in low and high frequency bands (Cuesta et al. 2022a, b; Ranasinghe et al. 2022; Schoonhoven et al. 2022). In particular, connectivity is often increased in the theta band, especially in the early disease stages, and decreased in alpha and higher bands (beta and gamma). In the present study we also observed differences between the results for the theta and the alpha band. For both types of connectivity measures, the best fit between empirical and model functional connectivity matrices tended to occur for relatively higher E/I balance for the theta band, and lower E/I balance for the alpha band. For the theta band, the best correlation was for positive values of the bifurcation parameter and a coupling strength around 1, whereas for the alpha band the best model fit was observed for negative values of bifurcation parameter *a* over a large range of G. This suggests that in general theta band connectivity could reflect dynamics in a more hyperexcited regime, while alpha band connectivity is associated with a more inhibited type of dynamics. This is of interest in view of the empirically observed increased connectivity in the theta band in AD patients, in particular those with subclinical epileptiform activity (Ranasinghe et al. 2022; Schoonhoven et al. 2022). The fact that empirical studies report a decreased connectivity in the alpha and higher bands might be related to the fundamentally different, more inhibitory dynamics in this frequency range.

However, we should point out that the relation between amyloid deposition, hyperexcitability, and local power changes in specific frequency bands is still the topic of active research. Nakamura et al. (2018) showed in a MEG study that amyloid deposition in the frontal regions may be related to increased power in the alpha band. Babiloni et al. (2022) did not find significant differences in local alpha power between 8 patients with MCI due to AD with epileptiform discharges in their EEGs compared to 34 patients without epileptiform discharges. In contrast, the subgroups with epileptiform discharges—as a signature of hyperexcitability—showed a significant increase in delta power in the occipital and temporal areas. Finally, a recent model study supports the relation between hyperexcitability and increased low frequency power (van Nifterick et al. 2022).

The primary aim of this study was to understand the relation between functional connectivity measures and E/I balance. In addition, we also explored whether our model has some potential to provide information on differences in E/I balance between subjects with subjective cognitive decline (SCD) and subjects with mild cognitive impairment (MCI). Here we expected to find evidence for a higher E/I balance as a sign of neural hyperexcitability in MCI as an early stage of Alzheimer’s disease. Compared to the contrasts between the two different types of connectivity measures, and the differences between the two frequency bands, the differences between the model fit of the SCD and MCI groups were relatively small. Furthermore, we have to take into account that we have only one single average FC matrix for each group per condition. Even so, the z-score plots of group differences in the empirical model correlation matrices do suggest some non-arbitrary patterns. Notably, for the AEC in the theta band, the SCD group seems to have a slightly better fit than the MCI group in the hyperexcitable region. The z-score plots of the other frequency bands and the phase coherence also suggest that both groups may have a characteristic “fingerprint” in terms of their optimal fit with the model in the excitation inhibition/coupling strength plane. A major challenge for future work is to extend this type of analysis to the individual level to obtain statistically supported conclusions about changes in excitation inhibition levels in early AD.

Other model studies have also investigated various aspects of functional connectivity and hyperexcitability in AD. In an early study we showed that disruption of synaptic strength induced by peak levels of synaptic activity could give rise to a transient phase of increased neural firings rates and interregional synchronization, followed by a late phase of spectral slowing, loss of activity and connectivity (de Haan et al. 2012). The present study replicates some of these findings in a much simpler model of the dynamics, where the E/I balance is compressed into a single parameter (the bifurcation parameter *a*). This simpler model greatly facilitates the investigation of amplitude and phase-based connectivity (which can both be derived directly from the model variables), and also enables fitting the model directly to empirical data, which is effectively impossible for the complex neural mass model used in other studies (de Haan et al. 2012; Stefanovski et al. 2019). Demirtas et al. (2017) used a whole brain model with Stuart Landau oscillators to explore the origins of decreased functional connectivity related to the progression of AD pathology. In this study empirical functional connectivity was based upon resting-state fMRI, and only showed a decrease going from healthy subjects to Alzheimer’s disease. Progressive loss of functional connectivity could be explained by successively lowering the bifurcation parameter from slightly negative to more negative values, further away from the equilibrium point at *a* = 0. These findings are more like those obtained in the alpha than the theta band in the present study, but direct comparison of fMRI and MEG findings is challenging. Of note, in a related study addressed specifically at modelling MEG resting state functional connectivity the authors showed that optimal results were obtained with a frequency of the oscillators of 12 Hz (Deco et al. 2017a; Tait et al. 2021) also used a modeling approach to explore hyperexcitability in AD. They used empirical, EEG-based functional connectivity to create individualized brain network models, on which they studied the propensity of these models to show a transition to seizure-like activity. This study showed a higher “brain network ictogenecity” (BNI) in the AD patients, with particular involvement of those brain areas that also typically display high levels of amyloid beta deposition. However, none of the AD patients in this study actually experience seizures, so the significance of the changes in BNI is not yet completely clear.

The present study has several limitations. First of all, the Stuart Landau model is relatively simple, and has no direct link to neurobiologically relevant features such as spike rates, membrane potentials, post synaptic potentials and time constants which feature prominently in more realistic neural mass models (de Haan et al. 2012; Haan et al. 2017; Stefanovski et al. 2019). The model generates steady state or oscillatory time series, but cannot deal with the 1/f part of the spectrum. However, this simplicity also has advantages since the behavior of this model is well understood, and it is easier to fit empirical data to his model and manipulate abstract features such as E/I balance. To keep the model simple, we did not include time delays between the oscillators. This approach without time delays was also used in another study that modeled MEG with Stuart Landau oscillators (Deco et al. 2017b). Please note that time delays are not necessary to obtain fluctuations of the amplitude envelope and amplitude envelope correlations in the model (see Fig. 3). We used a structural connectivity matrix based upon the positions of the centroids of AAL ROIs in combination with an exponential distance rule (Ercsey-Ravasz et al. 2013; Tzourio-Mazoyer et al. 2002). One might expect better results with structural networks based upon MRI tractography, but the exponential distance probably presents a very good approximation of the anatomical ground truth (Deco et al. 2021; Ercsey-Ravasz et al. 2013). The empirical data consisted of group level functional connectivity matrices averaged over all epochs of all subjects in a particular frequency band using either the AECc or the PLI. This was done to obtain FC matrices with the best possible signal-to-noise level for comparison with the model output. However, an important challenge for future studies is to explore the possibility of fitting the model to FC matrices of individuals rather than groups. Finally, the groups of SCD and MCI subjects were relatively small. Future studies should aim to study larger groups along the Alzheimer continuum to allow conclusions about disturbed E/I balance in different disease stages.

To conclude, we have shown that two different categories of functional connectivity measures based upon amplitudes or phases each have their own unique relation with the excitation E/I balance in the underlying brain networks. Furthermore, the relation between functional connectivity and excitation inhibition balance is modulated by the connection strength and the frequency band. These findings can guide the use of functional connectivity as biomarker of network hyperexcitability in AD, in particular when selecting subjects for future trials with anti-epileptic drugs. There are several challenges for future research. First of all, it would be helpful to explore new connectivity measures for their potential as network hyperexcitability biomarkers. One promising direction would be the concept of local and interregional neural variability (Garrett et al. 2013; Scheijbeler et al. 2022). Another challenge would be to fit models like the one used in this study to connectivity data of individual subjects. This would allow individual, quantitative assessment of the presence of network hyperexcitability, and could create new opportunities for therapeutic trials with anti-epileptic drugs of various forms of non-invasive stimulation.

## Supplementary Information

Below is the link to the electronic supplementary material.

Supplementary material 1 (TIFF 19780.0 kb)

Supplementary material 2 (TIFF 19780.0 kb)

## Data Availability

Data analyzed during the current study are not publicly available.

## Code Availability

BrainWave is freely available from website: home.kpn.nl/stam7883/brainwave.html.
