# Supplementary figures and images for: Network Hyperexcitability in Early Alzheimer’s Disease: Is Functional Connectivity a Potential Biomarker?
Source: Brain Topogr. 2023 May 12;36(4):595–612. doi: 10.1007/s10548-023-00968-7 (PMC10293463; doi:10.1007/s10548-023-00968-7)

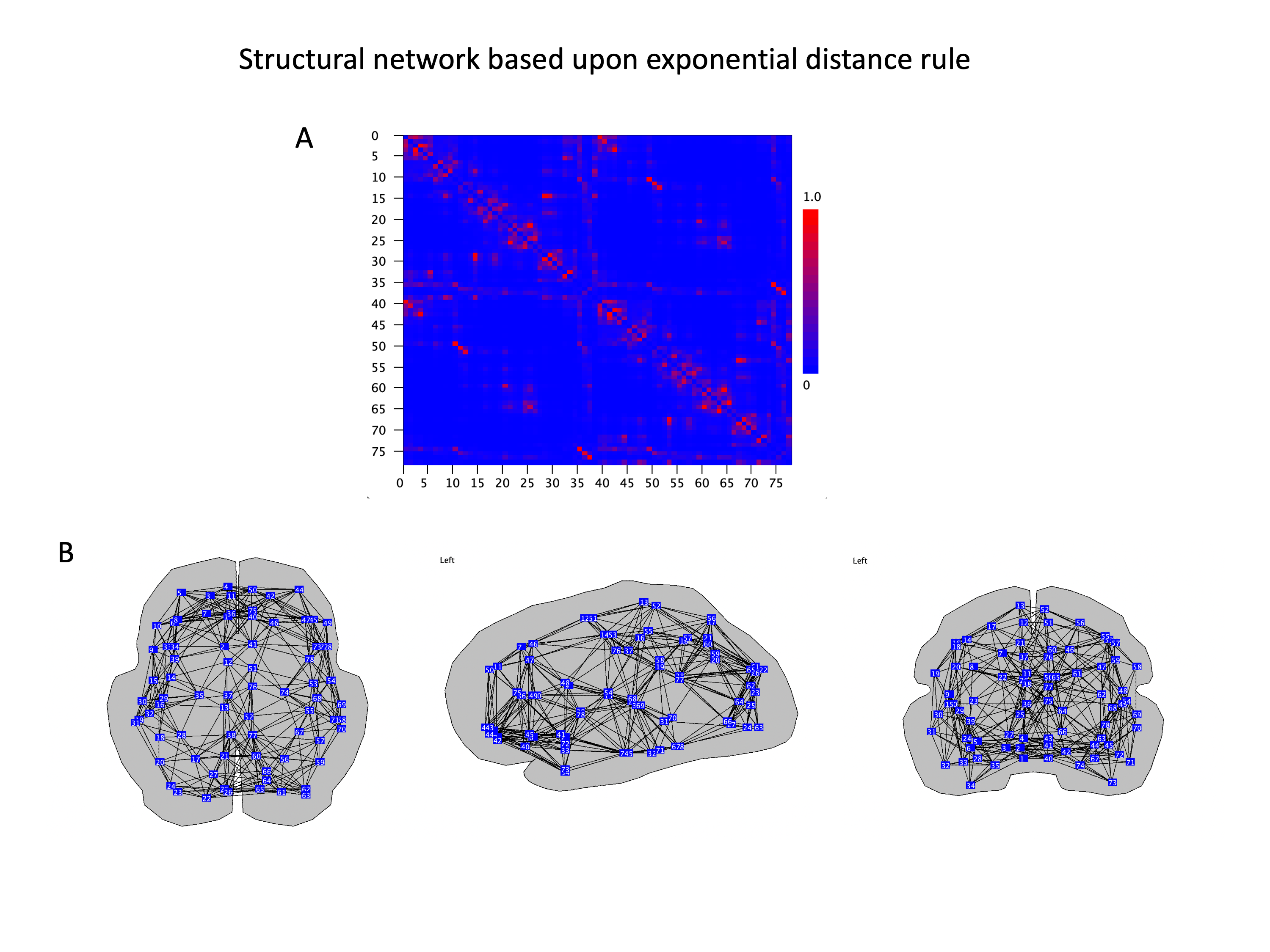

Supplement: Supplementary file 1 — Supplementary material 1 (TIFF 19780.0 kb) [file 10548_2023_968_MOESM1_ESM.tiff]

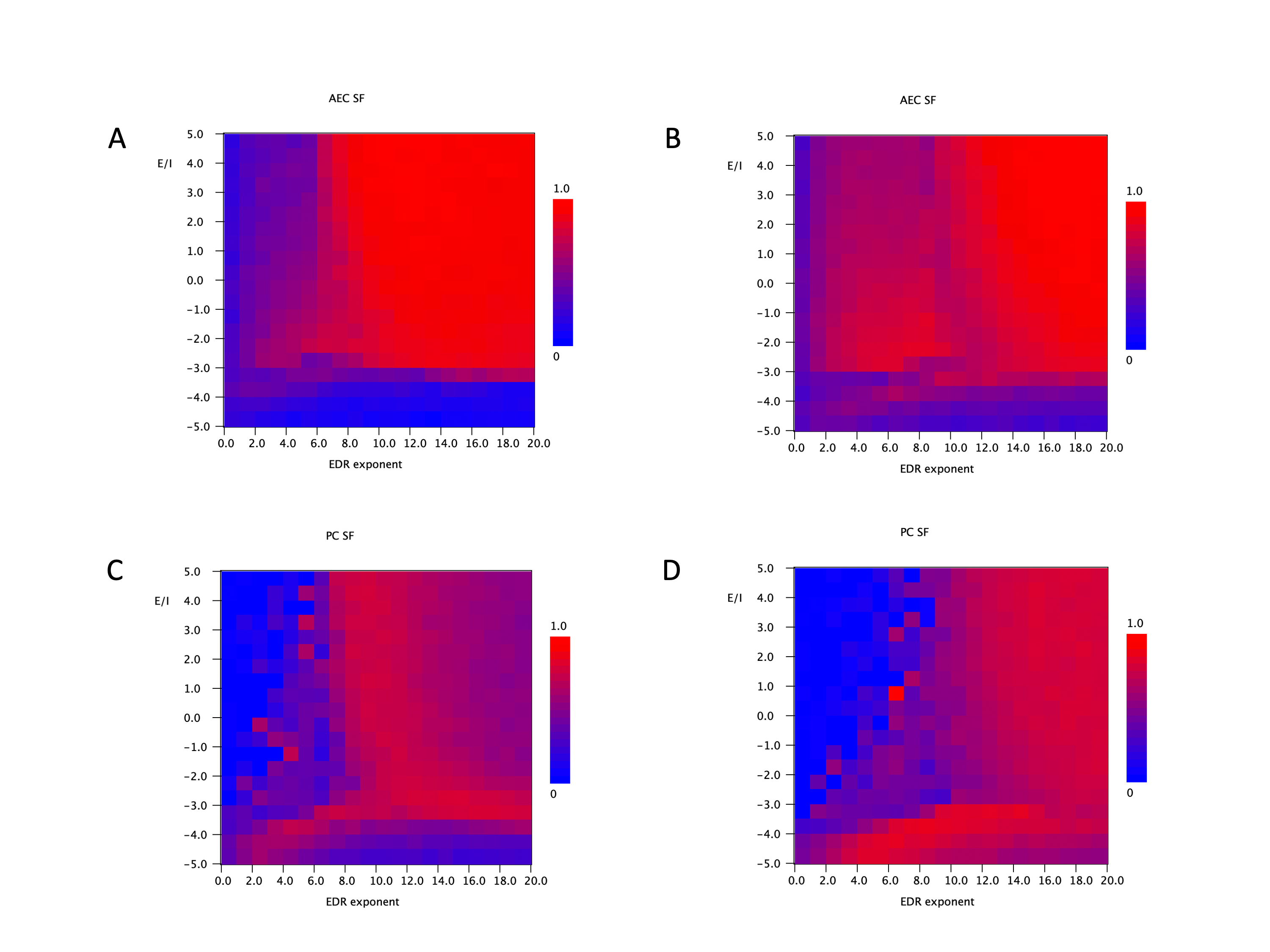

Supplement: Supplementary file 2 — Supplementary material 2 (TIFF 19780.0 kb) [file 10548_2023_968_MOESM2_ESM.tiff]
